# Fluvastatin Sodium Ameliorates Obesity through Brown Fat Activation

**DOI:** 10.3390/ijms20071622

**Published:** 2019-04-01

**Authors:** Na Yin, Hanlin Zhang, Rongcai Ye, Meng Dong, Jun Lin, Huiqiao Zhou, Yuanyuan Huang, Li Chen, Xiaoxiao Jiang, Kentaro Nagaoka, Chuanhai Zhang, Wanzhu Jin

**Affiliations:** 1 Key Laboratory of Animal Ecology and Conservation Biology, Institute of Zoology, Chinese Academy of Sciences, Beijing 100101, China; yinna@ioz.ac.cn (N.Y.); zhanghanlin@ioz.ac.cn (H.Z.); yerongcai@126.com (R.Y.); dongmeng90@foxmail.com (M.D.); cleverlinjun@126.com (J.L.); jessicachou36@126.com (H.Z.); lvhaizi@sina.com (Y.H.); 18811322312@163.com (L.C.); xiaoxiao0826@126.com (X.J.); 2 University of the Chinese Academy of Sciences, Beijing 100049, China; 3 Laboratory of Veterinary Physiology, Department of Veterinary Medicine, Tokyo University of Agriculture and Technology, Fuchu, Tokyo 183-8509, Japan; nagaokak@cc.tuat.ac.jp

**Keywords:** activator, brown adipose tissue, fluvastatin sodium, obesity

## Abstract

Brown adipose tissue (BAT), an organ that burns energy through uncoupling thermogenesis, is a promising therapeutic target for obesity. However, there are still no safe anti-obesity drugs that target BAT in the market. In the current study, we performed large scale screening of 636 compounds which were approved by Food and Drug Administration (FDA) to find drugs that could significantly increase uncoupling protein 1 (*UCP1*) mRNA expression by real-time PCR. Among those *UCP1* activators, most of them were antibiotics or carcinogenic compounds. We paid particular attention to fluvastatin sodium (FS), because as an inhibitor of the cellular hydroxymethyl glutaryl coenzyme A (HMG-CoA) reductase, FS has already been approved for treatment of hypercholesteremia. We found that in the cellular levels, FS treatment significantly increased *UCP1* expression and BAT activity in human brown adipocytes. Consistently, the expression of oxidative phosphorylation-related genes was significantly increased upon FS treatment without differences in adipogenic gene expression. Furthermore, FS treatment resisted to high-fat diet (HFD)-induced body weight gain by activating BAT in the mice model. In addition, administration of FS significantly increased energy expenditure, improved glucose homeostasis and ameliorated hepatic steatosis. Furthermore, we reveal that FS induced browning in subcutaneous white adipose tissue (sWAT) known to have a beneficial effect on energy metabolism. Taken together, our results clearly demonstrate that as an effective BAT activator, FS may have great potential for treatment of obesity and related metabolic disorders.

## 1. Introduction

Obesity, an increasing and serious worldwide problem, is mainly responsible for the development of type 2 diabetes and metabolic syndrome. Many studies have shown that obesity can induce a series of metabolic diseases, such as insulin resistance, type 2 diabetes, fatty liver, high cholesterol, high blood pressure, cardiovascular disease, polycystic ovary syndrome, inflammation, and cancer [1,2,3,4].

In contrast to energy-storing white adipose tissue (WAT), brown adipose tissue (BAT) burns energy through non-shivering thermogenesis. It has been shown that there is a negative relationship between body mass index and BAT [5,6]. Owing to the excellent energy expenditure ability of BAT, it is recognized as a promising target organ for obesity and diabetes therapy. Therefore, enhancing BAT activity and increasing BAT content may be effective therapeutic strategies to treat obesity-related diseases. Several studies have shown that BAT transplantation can be applied to treat obesity in mice [7,8,9,10], but this is not suitable for humans because of the immune-rejection and safety concerns. Hence, improving BAT activity via small molecules may be feasible for clinical application. The mitochondrial uncoupling protein 1 (*UCP1*), which is specifically expressed in BAT, can generate proton leakage in the mitochondrial inner membrane and then transfer energy to generate heat, which is a key marker of BAT activation. So far, cold stimulation [11], sympathetic input, catecholamine neurotransmission, thyroid hormones, cytokines, and small molecules, such as rutin [12], have been reported as activators of BAT [13,14,15]. Rutin is a BAT activator that we have recently discovered, whereas its low solubility and bioavailability limit its clinical application.

In this study, we used Food and Drug Administration (FDA)-approved natural products library to screen potential BAT activators that can be used to treat obesity. Among more than 600 small molecules, fluvastatin sodium (FS), which is used as a hypolipidemic drug, has received particular attention. FS is a member of the statin drug class used to treat hypercholesterolemia and prevent cardiovascular disease. The mechanism by which FS decreases serum cholesterol is blocking the liver enzyme 3-hydroxy-3-methylglutaryl-CoA (HMG-CoA) reductase, which facilitates cholesterol synthesis [16,17]. Administration of FS can reduce low-density lipoprotein (LDL) cholesterol as well as myocardial infarction to a similar degree as atorvastatin but a greater degree than rosuvastatin [18]. However, the relationship between FS and BAT has not been examined yet. In the current study, we investigated the effect of FS on BAT by both cellular and in vivo approaches. Our data show that FS plays an effective role in inhibiting obesity by activating BAT and may provide a new approach for dealing with metabolic disorders.

## 2. Results

### 2.1. FS Stimulates BAT Activity in Human Brown Adipocyte

To discover safe BAT activators, we screened 636 compounds that have already been approved by FDA for treating various diseases that could induce *UCP1* expression examined by qRT-PCR in human primary BAT cells.

**Figure 1:**
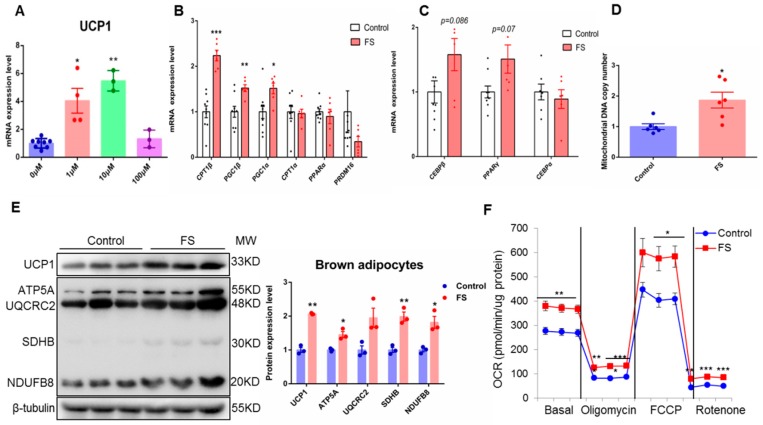
Fluvastatin sodium (FS) stimulates brown adipose tissue (BAT) activity in human brown adipocyte. (**A**) *UCP1* mRNA expression of differentiated human primary BAT cells treated by FS (0, 1, 10, or 100 μM) at day 6 for 24 h (*n* = 8, 4, 3 and 3), (**B**,**C**) fatty acid oxidation- and thermogenesis-related gene mRNA expression in differentiated human primary BAT cells treated by FS (10 μM, *n* = 6) or control (*n* = 9) at day 6 for 24 h, (**D**) mitochondrial DNA copy number of differentiated C3H10T1/2 cells treated by FS (10 μM) at day four for 48 h (*n* = 6), (**E**) oxidative phosphorylation-and thermogenesis-related protein expression of differentiated C3H10T1/2 cells treated by FS (10 μM) at day four for 48 h (*n* = 3), (**F**) oxygen consumption of differentiated C3H10T1/2 cells treated by FS (10 μM) at day four for 48 h (*n* = 5).Values represent means ± SEM. Error bars represent SEM; significant differences compared to vehicle controls are indicated by * *p* < 0.05, ** *p* < 0.01, *** *p* < 0.001 (assessed by Student’s *t*-test).

Among those *UCP1* inducers, most of them were antibiotics or carcinogenic compounds. We then focused on FS, which has less toxicity or side effects, for further investigations (Figure S1). Administration of FS could dose-dependently induce *UCP1* mRNA expression within 10 μM range, while at 100 μM concentration, we have observed cytotoxicity (Figure 1A). Therefore, in the subsequent experiment, 10 μM concentration was used as the optimal dosage. In concordant to the *UCP1* expression, the relative expression of fatty acid oxidation and thermogenesis-related genes (*PGC1α*, *PGC1β*, and *CPT1β*) were also significantly up-regulated by FS treatment (Figure 1B). Whereas adipogenic genes, such as *C/EBPα*, *C/EBPβ*, and *PPARγ*, did not show any changes (Figure 1C). BAT is characterized by abundant mitochondria, which are the main implementor of thermogenesis. Interestingly, we observed that FS significantly increased the mitochondrial DNA copy number (Figure 1D). Moreover, the expression of oxidative phosphorylation and thermogenesis-related proteins (UCP1 and OXPHOS) were also up-regulated by FS treatment (Figure 1E). Consistent with the above results, FS treatment significantly increased oxygen consumption (Figure 1F). Taken together, these results illustrate that FS can powerfully enhance BAT activity and mitochondrial functions without affecting brown adipogenesis at cellular levels.

### 2.2. FS Reduces Adiposity, Improves Glucose Homeostasis, and Increases Energy Expenditure in HFD Mice

**Figure 2:**
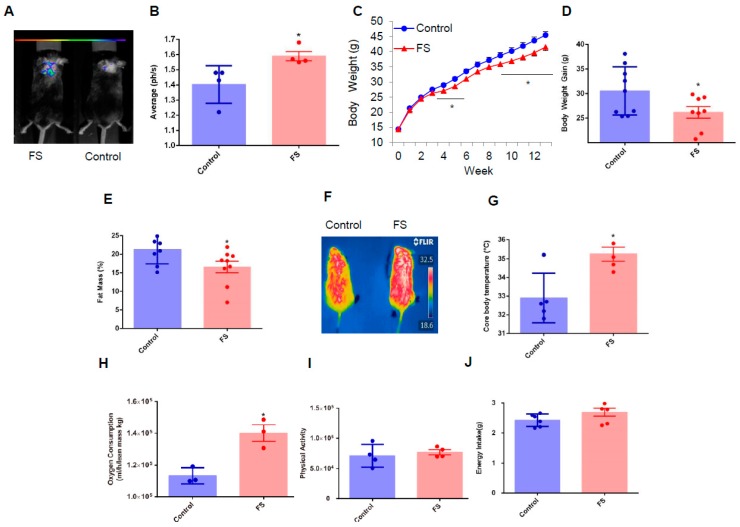
FS reduces adiposity and increases energy expenditure in HFD mice. (**A**,**B**) In vivo imaging of *UCP1* transgeneic luciferase male mice treated with saline or FS (1 mg/kg) for one week (*n* = 4), (**C**) body weight of C57/BL6 male mice treated by saline (*n* = 9) or FS (1 mg/kg, *n* = 8) fed with a high-fat diet from the fourth week, (**D**) body weight gain of C57/BL6 male mice treated with saline (*n* = 9) or FS (1 mg/kg, *n* = 8) in the 14th week, (**E**) fat mass of HFD male mice treated with saline (*n* = 8) or FS (1 mg/kg, *n* = 9) in the 11th week using NMR, (**F**,**G**) infrared imaging photos and core body temperature of HFD male mice treated with saline or FS (1 mg/kg) in a cold challenge experiment at 4 °C up to 4 h (*n* = 5), (**H**) oxygen consumption bar charts of mice treated with saline or FS in the 14th week for 48 h (*n* = 3), (**I**) physical activity of mice treated with saline or FS in the 14th week for 24 h (*n* = 4), (**J**) Average energy intake per day of mice treated with saline (*n* = 12) or FS (*n* = 10) in the 10th week for six days. Values represent means ± SEM. Error bars represent SEM. Significant differences compared to vehicle controls are indicated by * *p* < 0.05, ** *p* < 0.01 (assessed by Student’s *t*-test).

The above cellular evidence implies that FS might also have a beneficial effect on whole-body energy metabolism. To explore this possibility the *Ucp1*-luciferase transgenic mice [19] were used to assess whether FS can activate BAT. Excitingly, we found that FS substantially activated *UCP1* expression in BAT analyzed with a whole animal fluorescence imaging system (Figure 2A,B). Next, we investigated the effect of FS on body weight and whole-body energy metabolism in C57BL/6 mice fed with a high-fat diet from the age of four weeks. The FS treatment gradually but significantly decreased body weight gain from the fifth week and this effect was maintained till the end of the experimental period compared with the control group (Figure 2C,D). The whole-body fat mass was measured using non-radiotracer computerized tomography and showed that FS treatment can significantly decrease adipose tissue mass (Figure 2E and Figure S2A). In addition, these results were strongly supported by histological analysis which shows the size of the adipocytes from FS-treated mice was smaller than that of control mice (Figure S2B–D).

These beneficial effects were mediated by enhanced BAT activity as evidenced by results that FS treatment boosted core body temperature after cold stimulation (4 °C, 4 h, Figure 2F,G) and oxygen consumption (Figure 2H), without changes in physical activity and energy intake (Figure 2I,J). These results reveal that FS treatment effectively reduces adiposity and increases energy expenditure by significantly enhancing BAT activity in HFD mice.

### 2.3. FS Improves Glucose Homeostasis in HFD Mice

**Figure 3:**
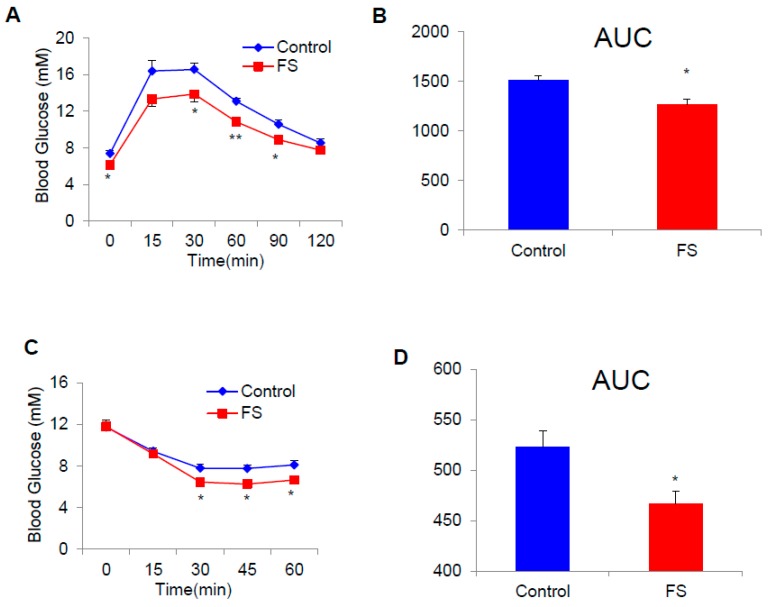
FS improves glucose homeostasis in HFD mice. (**A**,**B**) GTT and AUC (area under the curve) of HFD male mice treated with saline or FS (1 mg/kg) in the 13th week (*n* = 7), (**C**,**D**) ITT and AUC of HFD male mice treated with saline (*n* = 7) or FS (1 mg/kg, *n* = 6) in the 13th week. Values represent means ± SEM. Error bars represent SEM. Significant differences compared to vehicle controls are indicated by * *p* < 0.05, ** *p* < 0.01 (assessed by Student’s *t*-test).

Adiposity is often linked to the glucose homeostasis. We performed glucose tolerance tests (GTT) and insulin tolerance tests (ITT) to investigate the effect of FS on glucose homeostasis. The results indicate that FS could significantly improve glucose homeostasis (Figure 3A–D).

### 2.4. FS Increases Thermogenesis by Increasing BAT Activity

**Figure 4:**
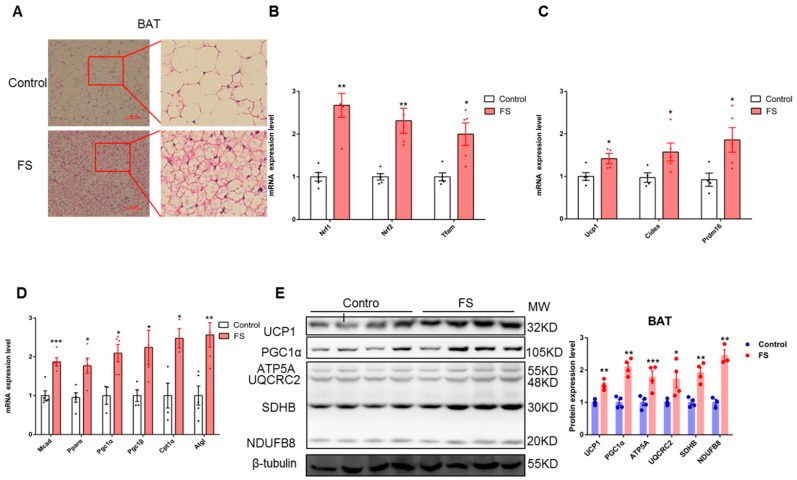
FS increases thermogenesis by increasing BAT activity. (**A**) BAT HE staining (200×, left) and enlarged graph (right) of mice treated with saline or FS, (**B**) relative expression (*n* = 5) of mitochondriogenesis-related genes (*Nrf1*, *Nrf2*, and *Tfam*), (**C**,**D**) relative expression (*n* = 5) of fatty acid oxidation- and thermogenesis-related genes (*Ucp1*, *Cidea*, *Prdm16*, *Mcad*, *Prarα*, *Pgc1α*, *Pgc1β*, *Cpt1α* and *Atgl*), **E**) expression of oxidative phosphorylation- and thermogenesis-related proteins (UCP1, PGC1α, and OXPHOS).Values represent means ± SEM. Error bars represent SEM. Significant differences compared to vehicle controls are indicated by * *p* < 0.05, ** *p* < 0.01, *** *p* < 0.001 (assessed by Student’s *t*-test).

To further explore the relationship between energy consumption and BAT activation, we investigated the morphological and molecular biological characteristics of BAT in mice after FS treatment. FS treatment reduced the size of lipid droplets in BAT as analyzed by H&E staining (Figure 4A) without alterations in BAT weight (Figure S2A). The expressions of mitochondriogenesis-related genes (*Nrf1*, *Nrf2*, and *Tfam*), fatty acid oxidation genes (*Mcad*, *Pparα*, *Pgc1α*, *Pgc1β*, *Cpt1α*, and *Atgl*), and thermogenesis-related genes (Ucp1, *Cidea*, and *Prdm16*) were significantly up-regulated by FS treatment in BAT (Figure 4B–D). Consistently, the expression of oxidative phosphorylation- and thermogenesis-related proteins (UCP1, PGC1a, and OXPHOS) was also significantly increased by FS treatment in BAT (Figure 4E). Taken together, these results indicate that FS treatment enhances endogenous BAT activity.

### 2.5. FS Relieves Hepatic Steatosis

**Figure 5:**
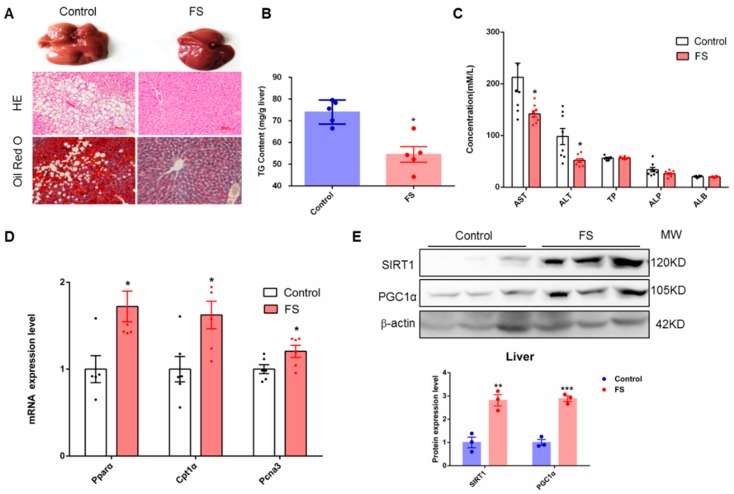
FS relieves hepatic steatosis. (**A**) Liver tissue (above), HE staining (middle), and Oil Red O staining (bottom) graphs of mice treated with saline or FS, (**B**) triglyceride (TG) contents in liver of mice treated with saline or FS (*n* = 5), (**C**) hepatic injury index in serum of mice treated with saline or FS (*n* = 8), (**D**,**E**) expression of liver-related genes (*Pparα*, *Cpt1α* and *Pcna3*) (*n* = 6) and proteins (SIRT1 and PGC1α).Values represent means ± SEM. Error bars represent SEM. Significant differences compared to vehicle controls are indicated by * *p* < 0.05 (assessed by Student’s *t*-test).

Hepatic steatosis is often associated with obesity. In the current study, we found that FS treatment notably ameliorated the hepatic steatosis induced by a high-fat diet as analyzed by histological examination and Oil Red O staining (Figure 5A) but did not affect liver weight (Figure S2A). In parallel, triglyceride content in the liver was significantly decreased in FS-treated mice (Figure 5B). Furthermore, FS treatment notably improved liver function without hepatotoxicity as analyzed by serum analysis (Figure 5C). As a hypolipidemic drug, FS indeed diminished LDL content in the serum (Figure S3). In addition, the expression level of fatty acid oxidation-related genes (*Pparα*, *Cpt1α*, and *Pcna3*) was increased in the liver from FS-treated mice (Figure 5D). Notably, FS treatment also significantly up-regulated the protein expression level of SIRT1 and PGC1α in the liver (Figure 5E), which suggests that FS may regulate liver fat metabolism through the SIRT1-PGC1α signaling pathway [20]. Altogether, these results demonstrate that FS can effectively ameliorate hepatic steatosis in HFD mice.

### 2.6. FS Increases Browning of sWAT

**Figure 6:**
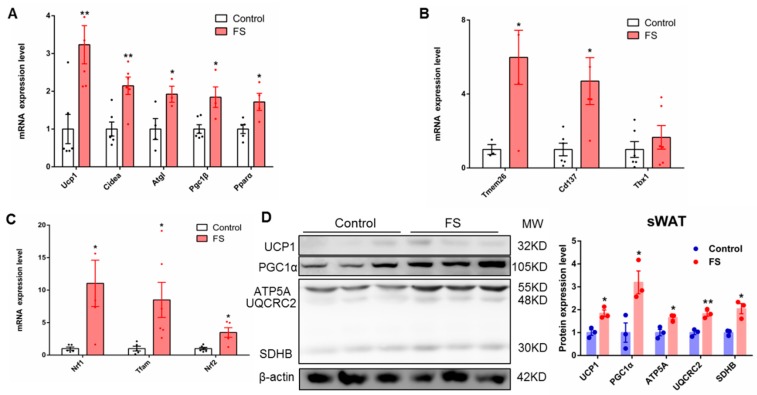
FS increases browning of sWAT. (**A**–**C**) Relative expressions (*n* = 6)of fatty acid oxidation- and thermogenesis-related genes (*UCP1*, *Cidea*, *Atgl*, *Pgc1β*, and *Pparα*), inflammatory factor genes (*Tmem26*, *Cd137*, and *Tbx1*), and relative expressions of mitochondriogenesis-related genes in sWAT (*Nrf1*, *Tfam*, and *Nrf2*), (**D**) the expression of oxidative phosphorylation-and thermogenesis-related proteins (UCP1, PGC1α, and OXPHOS).Values represent means ± SEM. Error bars represent SEM. Significant differences compared to vehicle controls are indicated by * *p* < 0.05, ** *p* < 0.01 (assessed by Student’s *t*-test).

Browning of sWAT can also improve energy metabolism, thereby has a beneficial effect on obesity. To investigate this possibility, we analyzed sWAT. We found that FS treatment significantly decreased the mass of sWAT and was associated with smaller adipocyte size (Figure S2A–C). Furthermore, results of the gene expression show that the expression of fatty acid oxidation- and thermogenesis-related genes (*UCP1*, *Cidea*, *Atgl*, *Pgc1β*, and *Pparα*) was significantly up-regulated in sWAT from FS-treated mice (Figure 6A). In addition, the genes which were expressed specifically in beige cells, including *Tmem26* and *Cd137*, were also notably up-regulated after FS treatment (Figure 6B). Moreover, the expressions of mitochondrial biogenesis-related genes (*Nrf1*, *Tfam*, and *Nrf2*) in sWAT were markedly increased after FS treatment (Figure 6C). Finally, the expression levels of oxidative phosphorylation- and thermogenesis-related proteins (UCP1, PGC1α, and OXPHOS) were significantly raised in sWAT from FS-treated mice (Figure 6D). These results suggest that FS treatment increased the browning of sWAT, which can be qualified as a synergistic effect with BAT activation to combat obesity.

## 3. Discussion

Our laboratory and other researchers have found that BAT transplantation can reverse metabolic disorders in various obese animal models [7,8,9,10,21]. These investigations clearly indicate that functional BAT is important in systemic energy metabolism. However, transplantation of BAT is not an easy way to apply in clinic. Therefore, increasing energy expenditure by enhancing BAT activity may be a feasible strategy against obesity [22]. Our laboratory has, therefore, been seeking safe and effective compounds to activate BAT. In this study, we found that FS is a novel and soluble efficient BAT activator after screening an FDA-approved drug library. Further study revealed that FS treatment could resist obesity, increase energy expenditure, maintain glucose homeostasis, improve insulin resistance, and ameliorate hepatic steatosis through activation of BAT and browning of sWAT. This is the first study to reveal the relationship between FS, BAT, and obesity.

FS is a statin that functions by reducing cholesterol levels in serum and is a popular drug for treating hypercholesteremia and cardiovascular diseases [23,24,25,26,27,28,29,30,31,32,33]. It has been reported that FS has an anti-obesity and cardioprotective effect [34]. Additionally, FS blunts the effects of a high-fat meal on plasma triglycerides and high-sensitivity C-reactive protein concentrations in patients at high risk for cardiovascular events [35]. Moreover, FS reduces the risk of major adverse cardiac events in diabetic patients [36], and very low-dose FS decreases inflammation and oxidative stress in patients with type 1 diabetes. In addition, FS ameliorates the polyuria associated with X-linked nephrogenic diabetes insipidus in mice [37]. However, some researchers have found that FS is associated with an increased incidence of type 2 diabetes, impairs insulin signaling, and worsens insulin resistance, inducing inflammatory responses by the NLRP3/caspase-1 pathway [38]. Furthermore, FS increases body and liver fat accumulation in a model of metabolic syndrome and increases subcutaneous fat deposition [39]. These differences in the results after FS treatment may be due to the different animal models and doses used in researches. Moreover, this is a reasonable phenomenon, because the pharmacological effects of drugs are complex, there are too many unknown effects under different backgrounds worth exploring. As fluvastatin, lovastatin, simvastatin, and pravastatin are stains, the *UCP1* mRNA expression (5.69 vs. 0.65, 1.45, and 0.27) in human brown adipocytes is different in different stains. It suggests that the other stains do not have the same effect as fluvastatin. This experiment does not indicate that inhibition of HMG-CoA reductase contributes to browning. Altogether, FS can reduce cholesterol levels in serum, has anti-obesity and antioxidant stress effects, decreases inflammation, and ameliorates diabetes. This is consistent with our results showing that FS treatment inhibits obesity, improvs diabetes, and reduces LDL levels in serum. This is the first time that these effects of FS have been linked to the functionality of BAT.

Our in vitro results show that FS enhances brown adipocyte activity and mitochondrial function in human brown adipocytes (Figure 1). These results demonstrate that FS can directly activate brown adipocytes. Our in vivo results show that FS significantly reduces adiposity and body weight gain by enhancing energy expenditure and improves glucose homeostasis in HFD mice. We also found that FS treatment markedly increases the expression of UCP1 at both the mRNA and protein levels, as well as the expression of thermogenic genes and OXPHOS protein.

Interestingly, we also found that FS ameliorates hepatic steatosis and significantly reduces the hepatic injury induced by a high-fat diet, which is in contrast to previous research [39]. This may be due to the differences in experimental conditions and animal models. Finally, we found that FS treatment promotes the expression of thermogenic genes and related proteins, especially UCP1, in sWAT, in which browning was induced after FS treatment. This is similar to the effect of rutin on sWAT as we reported previously [40]. Decreased lipid synthesis in sWAT and liver may also contribute significantly to the anti-obesity effect and improve energy metabolism. One of the limitations of our study is that we did not explore the underlying mechanism of how FS up-regulates UCP1 in BAT and sWAT, which is a worthy subject for future study.

In summary, for the first time, we found that FS can activate BAT to reduce obesity. Although the types of drugs and compounds we screened were not comprehensive enough, we may further improve and expand our screening range to establish a more complete research system in the future. Collectively, our results show that FS prevents obesity in HFD mice partly through activating BAT and recruiting beige cells in sWAT. Further detailed study of FS, especially its underlying mechanisms, may generate new ideas for dealing with obesity or maintaining energy metabolism in the body.

## 4. Materials and Methods

### 4.1. Animals

C57BL/6 male mice (four-weeks-old) were obtained from Beijing Vital River Laboratory Animal Technology. ThermoMouse: *Ucp1-Luc2-tdTomato* Kajim/J mice were obtained from the Jackson Laboratory, Stock No: 026690 (https://www.jax.org/strain/026690), detailed information can be obtained from the article [19]. Five mice per cage were housed under constant environmental conditions in an office of Laboratory Animal Welfare-certified animal facility with a 12-h light-dark cycle. Water and food were provided ad libitum. All of the animal studies were conducted with the approval (IOZ20160028) of the Institutional Animal Care and Use Committee of the Institute of Zoology, Chinese Academy of Sciences.

### 4.2. Fluvastatin Sodium Treatment

FS (from Shanghai Bide Pharmatech Ltd., BD23193, 98%) was dissolved in sterile 0.9% NaCl normal saline for mouse experiments. Males were randomly assigned by body weight and animals were fed with a high-fat diet (HFD) at arrival in the laboratory. Male mice were orally treated with FS (1 mg/kg body weight per day) or saline while fed with HFD. HFD (D12492i) contained 60 kcal% fat (Research Diets, New Brunswick, NJ, USA). The body weight was tested weekly, and the energy intake was tested at the tenth week for continuous six days. The fat mass and lean mass were measured using a nuclear magnetic resonance (NMR) instrument in the 11th week. The mice were put into a physical activity instrument to record their movements. Oxygen consumption was measured by a TSE lab master system, as described previously [11]. Mice were placed in cages for 48 h, and then VO_2_ and VCO_2_ were measured during the next 48 h. Mice were maintained at 24 °C under a 12-h light/dark cycle with free access to food and water during measurements. All the animal experiments were performed on the same batch of mice. The physical activity experiment (*n* = 4) and the cold challenge experiment (*n* = 5) were performed at the same time.

### 4.3. Cell Experiments

FS was dissolved in sterile distilled deionized water for cell treatment. Human primary BAT cells (gifted from the Beijing Luhe Hospital Capital Medical University) [41] were cultivated in Dulbecco’s modified Eagle medium (DMEM) (high glucose, 450 mg/dL) containing 20% fetal bovine serum (FBS) (*v*/*v*), 20 mM HEPES, penicillin, and streptomycin. The human differentiation medium contained 2% FBS (*v*/*v*), 0.5 mM isobutylmethylxanthine, 0.125 mM indomethacin, 33 μM biotin, 17 μM pantothenic acid, 2 nM triiodothyronine, 0.5 μM insulin, 2 μg/mL dexamethasone, penicillin, and streptomycin. Human primary BAT cells were cultivated in human differentiation medium for seven days, then treated with different doses of FS for 24 h. C3H10T1/2 cells were cultivated in the DMEM (high glucose, 450 mg/dL) containing 10% FBS (*v*/*v*), penicillin, and streptomycin. Mouse induction medium contained 10% FBS (*v*/*v*), 0.5 mM isobutylmethylxanthine, 0.125 mM indomethacin, 1 nM triiodothyronine, 20 nM insulin, 2 μg/mL dexamethasone, penicillin, and streptomycin. The mouse differentiation medium contained no isobutylmethylxanthine, indomethacin, or dexamethasone. C3H10T1/2 cells were cultivated in the mouse induction medium for two days, and then in mouse differentiation medium for four days. C3H10T1/2 cells were treated with different doses of FS on the sixth day for 24 h. C3H10T1/2 cells were treated with or without FS (10 μM) for six days during brown adipogenesis. O_2_ consumption of fully differentiated adipocytes was measured at day six with an XF24-3 extra cellular flux analyzer (Agilent Technologies, Santa Clara, CA, USA). Basal respiration was also assessed in untreated cells.

### 4.4. Compound Screening

Human primary BAT cells were cultivated in Dulbecco’s modified Eagle medium (DMEM) (high glucose, 450 mg/dL) containing 20% fetal bovine serum (FBS) (*v*/*v*), 20 mM HEPES, penicillin, and streptomycin. Human primary BAT cells were treated with different compounds for 24 h at 1 μM.

### 4.5. Real-Time Quantitative PCR

Cells in 12-well plates were washed by phosphate buffered saline (PBS) three times, and then were given 0.5 mL TRIZOL reagent (Invitrogen, Carlsbad, USA). Tissues were pulverized and then given 1 mL TRIZOL reagent. Total RNA was extracted using the TRIZOL method. RNA (2 μg) was reverse transcribed to cDNA using a high-capacity cDNA reverse transcription kit (Promega Biotech, Madison, WI, USA). Gene expression was tested using real-time quantitative PCR analysis (ABI Prism VIIA7; Applied Biosystems) performed with a SYBR Green Master Mix (Promega Biotech, Madison, WI, USA) and normalized by cyclophilin expression. Primers were designed using Primer Quest (Integrated DNA Technologies, Coralville, IA, USA). Primer sets are described in Table 1.

### 4.6. Western Blotting

Cells in 12-well plates were washed by PBS three times, and then were given 100 μL RIPA reagent (1.0% Triton X-100, 150 mM sodium chloride, 0.5% sodium deoxycholate, 0.1% SDS, 50 mM Tris) containing phosphatase inhibitor and protease inhibitor (Roche Diagnostics, Rotkreuz, Switzerland). Tissues were pulverized, and then dissolved in 200 μL RIPA reagent containing phosphatase inhibitor and protease inhibitor. Protein was collected by centrifugation for 10 min at 4 °C and 13,000 rpm. The protein concentration was measured by a BCA assay kit (Pierce Diagnostics). Protein of 30 μg was mixed with 5× loading buffer. Protein samples were heated for 10 min at 95 °C and put on ice immediately. Protein was separated by 10% (*w*/*v*) SDS/PAGE (40 min at 70 V in a stacking gel, then 1 h at 120 V in the spacer gel of the electrophoresis apparatus), transferred for 2.5 h at 100 V and 200 mA to a PVDF membrane (Millipore, Burlington, MA, USA, blocked in 5% (*w*/*v*) skim milk (OXOID) in TBST (0.02 M Tris base, 0.1% Tween 20, 0.14 M NaCl pH 7.4), incubated by primary antibodies overnight at 4°C, and then incubated with secondary antibodies conjugated with horseradish peroxidase for 1 h at room temperature. The following are the primary antibodies used in this study: anti-UCP1 (1:1000; Abcam, Cambridge, MA, USA, ab155117 [42] and ab10983 [43]), anti-OXPHOS (1:250; Abcam, ab110413 [44]), anti-PGC1α(1:1000; Abcam, ab54481 [45]), anti-β-Tubulin (1:1000; Santa Cruz biotechnology, Dallas, TX, USA, SC-9014 [46]), Sirt1(1:500; cell signaling technology, Danvers, MA, USA, 8469 [47]), and anti-β-actin (1:1000; Sigma-Aldrich, St. Louis, MO, USA, A2228 [48]). Signals were detected with Supersignal West Pico Chemiluminescent Substrate (Pierce, Waltham, MS, USA).

### 4.7. Glucose Tolerance Test and Insulin Tolerance Test

For glucose tolerance test (GTT), the mice were fasted for 16 h from the 5:00 pm to the next day at 9:00 am with free access to drinking water. Fasting blood glucose and body weight were recorded before mice were intraperitoneally injected with 10% D-glucose (*w*/*v*) (1.0 g/kg body weight). Blood glucose levels were measured at 15, 30, 60, 90, and 120 min after D-glucose injection with an Accu-Chek glucose monitor (Roche Diagnostics Corp, Rotkreuz, Switzerland). After three days, for the insulin tolerance test (ITT), the mice were fasted for 4 h (08:00–12:00) with free access to drinking water, then intraperitoneally injected with insulin (1 U/kg body weight) (Humulin; Eli Lilly, Indianapolis, IN, USA). Blood glucose levels were measured before and at 15, 30, and 60 min after insulin injection.

### 4.8. In Vivo Imaging

As only male mice are carriers of the transgene, the transgenic insertion site is most likely on the Y chromosome. *Luciferase 2* and *td-Tomato* fusion genes are expressed in brown adipose tissue and beige cells. The transgenic male mice were mated with C57BL/6J female mice to obtain transgenic male offspring. The sixth-generation mice had been bred back to the C57BL/6J background. Sixth-generation transgenic male mice were placed on a chow diet and were orally treated with FS (1 mg/kg) for one week. Luciferase activity was monitored using a bio-analytical instrument (Berthold Technologies, Bad Wildbad, Germany). Images (Photo: x-Binning:1, y-Binning:1; Luminescence: x-Binning:8, y-Binning:8; exposure time: 300 s) were collected starting 10 min after injection of 150 mg/kg luciferin substrate (Goldbio, St. Louis, MO, USA), while the mice were anaesthetized using 4% isoflurane. Luciferase activity was calculated using Indigo Software (Berthold Technologies, Bad Wildbad, Germany).

### 4.9. Cold Challenge Experiment

The mice were put into a 4 °C refrigerator for up to 4 h with free access to food and water. The core temperatures of mice at 0, 1, 2, 3 and 4 h were recorded using a rectal probe connected with a digital thermometer (Yellow Spring Instruments, Yellow Springs, OH, USA). Infrared images were taken at 4 h using an infrared digital thermographic camera (E60: Compact Infrared Thermal Imaging Camera; FLIR Systems, Wilsonville, OR, USA) and analyzed by FLIR Quick Report software (FLIR ResearchIR Max 3.4; FLIR Systems).

### 4.10. Hematoxylin-Eosin (H&E) Staining

Tissues were fixed in PBS buffer containing 4% paraformaldehyde overnight at room temperature and washed on a shaking table at room temperature. Then the tissues were stained according to standard protocols. Resin and cover glass were used to seal the section. Images were taken by an inverted microscope (DS-RI1; Nikon, Tokyo, Japan).

### 4.11. Oil Red O Staining

We dissolved 0.5 g Oil Red O (Oil Red O; Sigma, catalog#0-0625) in 100 mL isopropanol staining stock. Oil Red O staining buffer consisted of 6 mL Oil Red O staining stock mixed with 4 mL ddH_2_O, used after filtration and prepared when needed. Sections of 5-μm thickness were stained with Oil Red O staining buffer for 1 h, washed with ddH_2_O five times, and dried in air. The results were observed by an inverted microscope (DS-RI1; Nikon).

### 4.12. Measurement of Mitochondrial DNA Copy Number

Total DNA (genomic and mitochondrial DNA) was extracted from the differentiated human primary BAT cells using a QIA amp DNA Mini kit (Qiagen, Venlo, Netherlands) according to the manufacturer’s instructions. DNA concentrations were assessed using a Nanodrop 2000 (Thermo Scientific, Wilmington, DE, USA). The mitochondrial DNA (mtDNA) copy number relative to genomic DNA content was quantitatively analyzed using an ABI Prism VIIA7 real-time PCR (Applied Biosystems, Waltham, MA, USA). Primer sequences for *COX II* and β globin were as follows: *COX II*: forward GCCGACTAAATCAAGCAACA, reverse CAATGGGCATAAAGCTATGG, β globin: forward GAAGCGATTCTAGGGAGCAG, reverse GGAGCAGCGATTCTGAGTAG.

### 4.13. Statistical Analysis

All of the results are expressed as means ± standard deviation (SD) using Prism software. To test normality, the Shapiro–Wilk test was performed, and then, depending on its outcome, data were analyzed using Student’s *t*-test or one-way ANOVA with Turkey’s post hoc tests. Statistical significance was set at *p* < 0.05.

## Figures and Tables

**Table 1:** Primer sequences.

Gene	Forward Primer (5′→3′)	Reverse Primer (5′→3′)
*Human-Cyclophilin*	TAAAGCATACGGGTCCTGGC	GACTGAGTGGTTGGATGGCA
*Human-UCP1*	GCAGGGAAAGAAACAGCACC	CCCGTGTAGCGAGGTTTGAT
*Human-PGC1α*	CAGGCAGTAGATCCTCTTCAAG	TCCTCGTAGCTGTCATACCTG
*Human-PGC1β*	GCCCAGATACACTGACTACG	CTCGAGGGTTAAGGCTGTTATC
*Human-PPARα*	GCTATCATTACGGAGTCCACG	TCGCACTTGTCATACACCAG
*Human-PPARγ*	ATACATAAAGTCCTTCCCGCTG	GGGTGATGTGTTTGAACTTGATT
*Human-PRDM16*	TTCGGATGGGAGCAAATACTG	CACGGATGTACTTGAGCCAG
*Human-CPT1β*	ATCCTACTCCTATGACCCCG	TCTGCATTGAGACCCAACTG
*Human-CPT1α*	CCTCCAGTTGGCTTATCGTG	TTCTTCGTCTGGCTGGACAT
*Human-CEBP/α*	ACTAGGAGATTCCGGTGCCT	GAATTCTCCCCTCCTCGCAG
*Human-CEBP/β*	GCACAGCGACGAGTACAAGA	TTGAACAAGTTCCGCAGGGT
*Mouse-Cyclophilin*	CAAATGCTGGACCAAACACAA	GCCATCCAGCCATTCAGTCT
*Mouse-Ucp1*	GGCAAAAACAGAAGGATTGC	TAAGCCGGCTGAGATCTTGT
*Mouse-Cidea*	TGCTCTTCTGTATCGCCCAGT	GCCGTGTTAAGGAATCTGCTG
*Mouse-Prdm16*	GAAGTCACAGGAGGACACGG	CTCGCTCCTCAACACACCTC
*Mouse-Cpt1α*	GACTCCGCTCGCTCATTCC	GACTGTGAACTGGAAGGCCA
*Mouse-Mcad*	ACTCGAAAGCGGCTCACAA	ACGGGGATAATCTCCTCTCTGG
*Mouse-Pgc1α*	ACAGCTTTCTGGGTGGATTG	TGAGGACCGCTAGCAAGTTT
*Mouse-Pgc1β*	CGTATTTGAGGACAGCAGCA	TACTGGGTGGGCTCTGGTAG
*Mouse-Pparα*	AGCCTCAGCCAAGTTGAAGT	TGGGGAGAGAGGACAGATGG
*Mouse-Tbx1*	GGCAGGCAGACGAATGTTC	TTGTCATCTACGGGCACAAAG
*Mouse-Tmem26*	ACCCTGTCATCCCACAGAG	TGTTTGGTGGAGTCCTAAGGTC
*Mouse-Cd137*	CGTGCAGAACTCCTGTGATAAC	GTCCACCTATGCTGGAGAAGG
*Mouse-Atgl*	GCGCCAGGACTGGAAAGAAT	TGAGAACGCTGAGGCTTTGAT
*Mouse-Tfam*	GTCCATAGGCACCGTATTGC	CCCATGCTGGAAAAACACTT
*Mouse-Nrf1*	CAACAGGGAAGAAACGGAAA	GCACCACATTCTCCAAAGGT
*Mouse-Nrf2*	CCCCCGAGGACACTTCTTATG	AGCAGCCAGATGGGCAGTTA
*Mouse-Pcna3*	CACGTCTCCTTGGTACAGCTTACTC	CACGCCCATGGCTAGGTT

## Supplementary Materials

Supplementary materials can be found at https://www.mdpi.com/1422-0067/20/7/1622/s1. Figure S1. Compound screen by qRT-PCR; Figure S2. organ weight and immunohistochemical index; Figure S3. Plasma lipid profiles.
