# Supplementary material for: Fluvastatin Sodium Ameliorates Obesity through Brown Fat Activation
Source: Int J Mol Sci. 2019 Apr 1;20(7):1622. doi: 10.3390/ijms20071622 (PMC6479292; doi:10.3390/ijms20071622)
Supplement: Supplementary file 1 [file ijms-20-01622-s001.zip › supplementary figures.pdf]

Supplementary figures

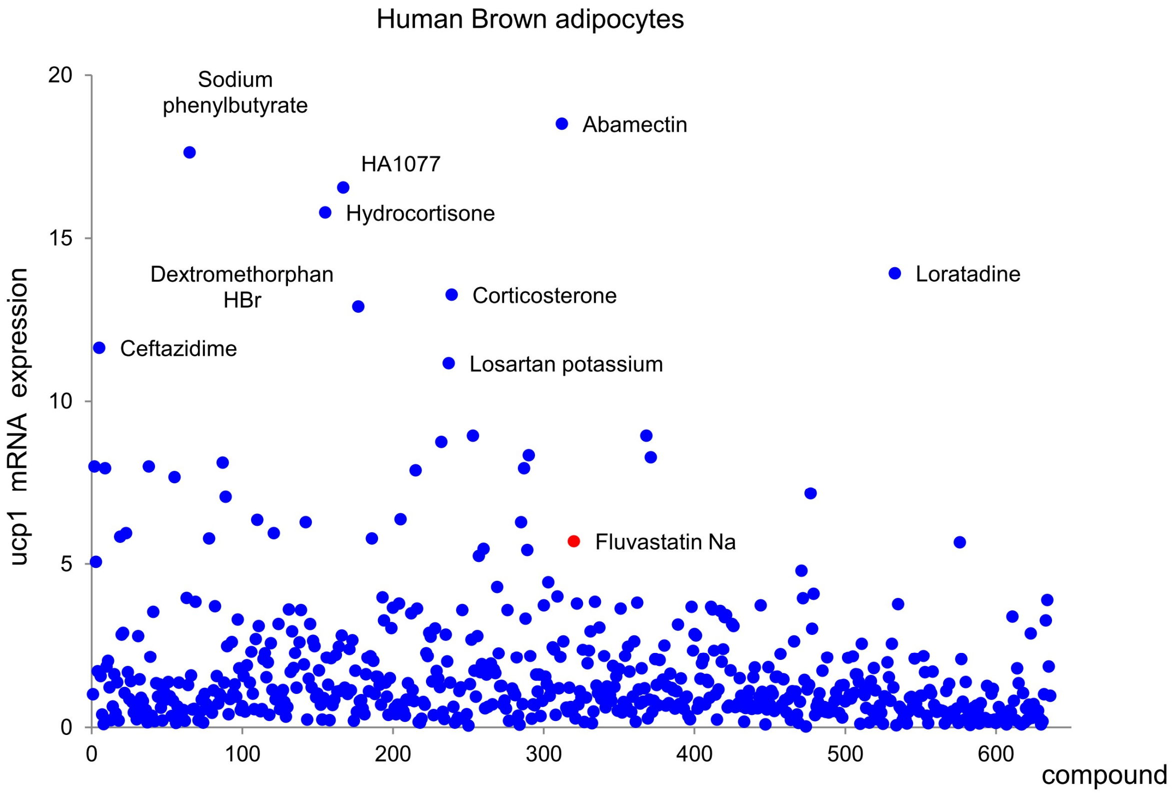

Figure s1. Compound screen by qRT-PCR.

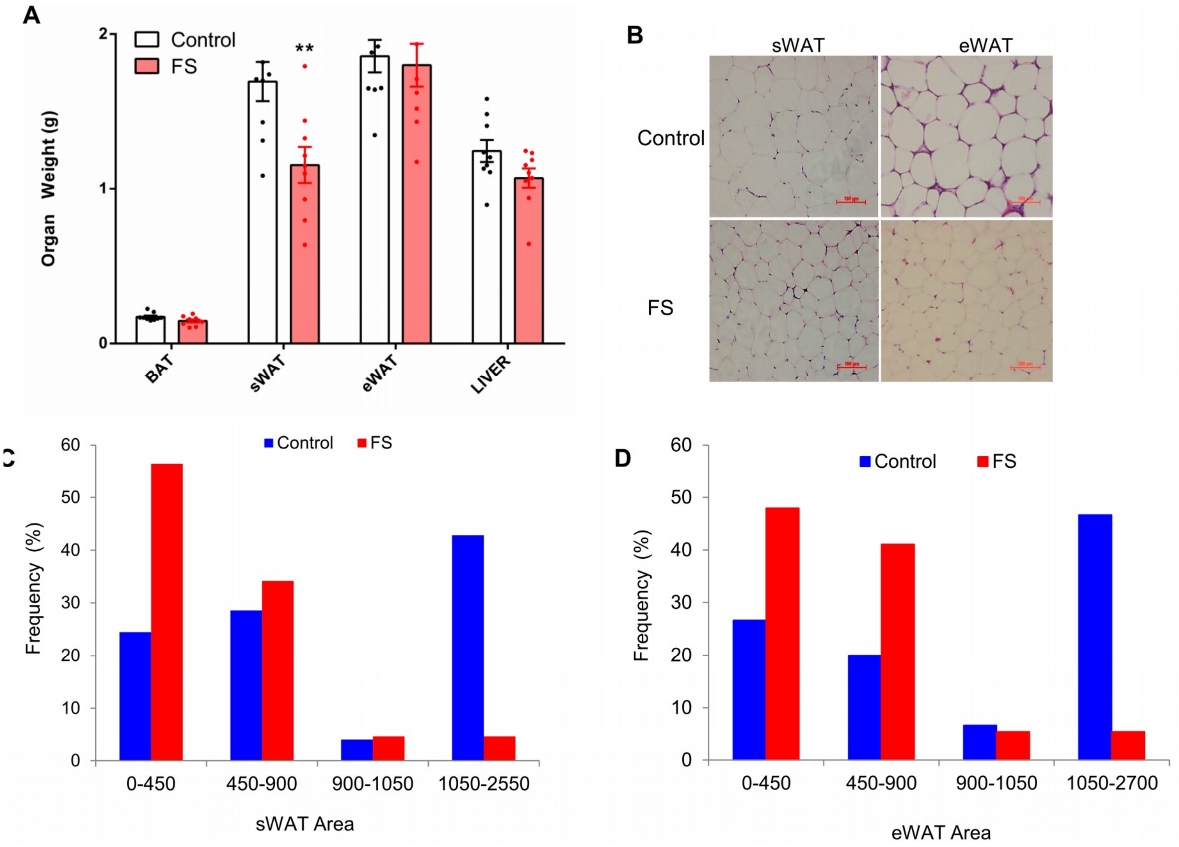

Figure S2. organ weight and immunohistochemical index.

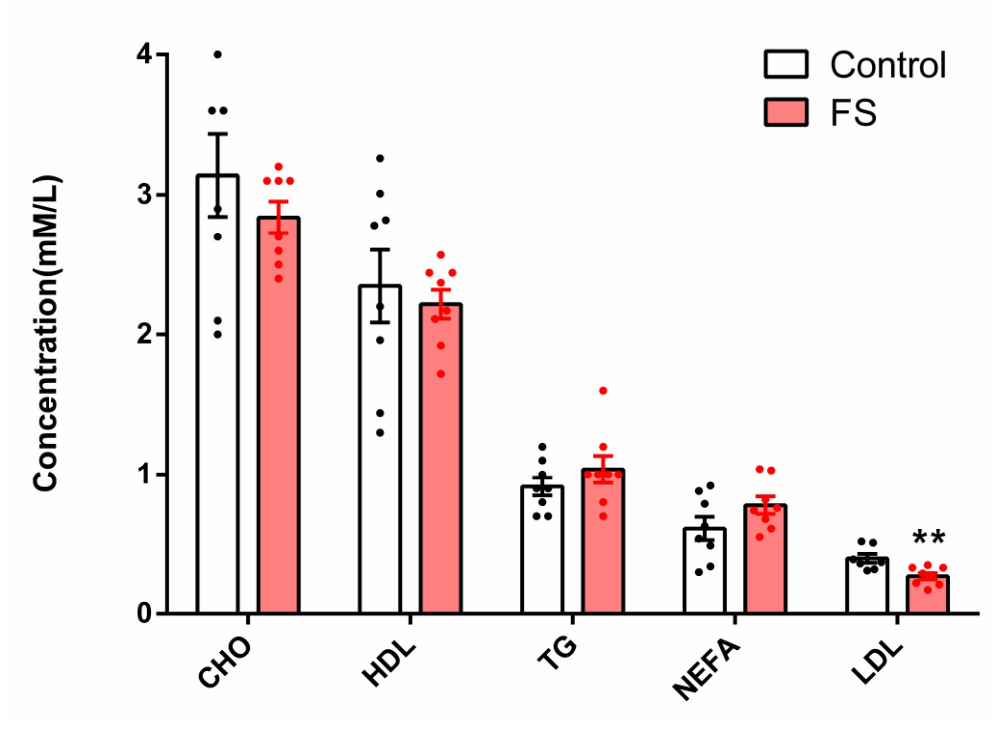

Figure s3. Plasma lipid profiles.
